# Biodegradation of different formulations of polyhydroxybutyrate films in soil

**DOI:** 10.1186/s40064-016-2480-2

**Published:** 2016-06-17

**Authors:** Nadia Altaee, Gamal A. El-Hiti, Ayad Fahdil, Kumar Sudesh, Emad Yousif

**Affiliations:** Department of Biotechnology, College of Science, Al-Nahrain University, Baghdad, 10001 Iraq; Department of Horticulture and Garden Engineering, College of Agriculture, Al-Qasim Green University, Babil, Al-Qasim 51002 Iraq; Cornea Research Chair, Department of Optometry, College of Applied Medical Sciences, King Saud University, P.O. Box 10219, Riyadh, 11433 Saudi Arabia; School of Biological Sciences, Universiti Sains Malaysia, 11800 Penang, Malaysia; Department of Chemistry, College of Science, Al-Nahrain University, Baghdad, 64021 Iraq

**Keywords:** Polyhydroxyalkanoates, Polyhydroxybutyrate, Electrospinning, Biodegradation, Nanofibers, UV treatment

## Abstract

**Background:**

Petroleum polymers contribute to non-degradable waste materials and it would therefore be desirable to produce ecofriendly degradable materials. Biodegradation of polyhydroxybutyrate (PHB) in the presence of oligomer hydrolase and PHB depolymerase gave 3-hydroxybutyric acid which could be oxidized to acetyl acetate. Several bacteria and fungi can degrade PHB in the soil.

**Results:**

Biodegradation of PHB showed a significant decrease in the molecular weight (Mw), number-average molecular weight (Mn) and the dispersity (Mw/Mn) for all the film formulations. Nanofibers of PHB and its composites showed faster degradation compared to other films and displayed complete degradation after 3 weeks. The SEM micrographs showed various surface morphology changes including alterations in appearance of pores, cavity, grooves, incisions, slots and pointers. Such changes were due to the growth of microorganisms that secreted PHB depolymerase enzyme which lead to the biopolymer films degradation. However, PHB nanofibers and its composites films in the presence of TiO_2_ demonstrated more surface changes with rupture of most nanofibers in which there was a drop in fibres diameter.

**Conclusions:**

The degradation of biopolymers help to overcome some of the pollution problems associated with the use of petroleum polymers. PHB nanofiber and its TiO_2_ composite were degraded faster compared to other PHB film types due to their three dimensional and high surface area structures. The presence of TiO_2_ nanoparticles in the composite films slowdown the degradation process compared to PHB films. Additionally, the PHB and its composite films that were prepared from UV treated PHB films led to acceleration of the degradation.

**Graphical abstract:**
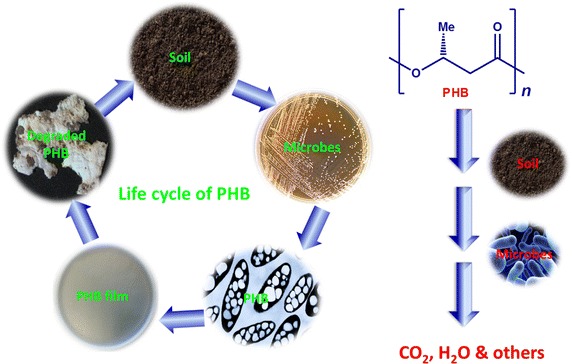
Biodegradation of polyhydroxybutyrate films in soil

## Background

The biodegradation process is a biological activity of living organisms to decompose the complex structure of organic compounds to nontoxic products with lower molecular weights. The end products of the biodegradation process can be used as an energy and nutritional source for anabolism of non-producing organisms (Braunegg et al. 1998). The biodegradation of polyhydroxyalkanoates (PHAs) takes place either under anaerobic conditions to produce carbon dioxide (CO_2_), water (H_2_O) and methane or under aerobic conditions to produce CO_2_ and H_2_O (Wang et al. 2013, 2014; Gutierrez-Wang et al. 2010; Mueller 2006; Avella et al. 2005; Jendrossek and Handrick 2002; Abou-Zeid et al. 2001). Biodegradation of PHAs can also occur within the cellular cytoplasm by intracellular depolymerase which is commonly referred to intracellular biodegradation (Mergraet et al. 1992). Also, biodegradation by extracellular depolymerase in the surrounding environment is known as extracellular biodegradation (Mergraet et al. 1992). Many factors such microbial activity, polymer composition, molecular weight, crystallinity, temperature, moisture, pH, nutrient content and oxygen can affect the biodegradation process (Boopathy 2000; Bernard 2014). In addition, the surface area of polymeric materials can have an effect on the biodegradation rate where a lower surface area can restrict the microbial growth (Tokiwa et al. 2009).

Polyhydroxybutyrate (PHB) can be degraded to 3-hydroxybutyric acid by oligomer hydrolase and PHB depolymerase. The 3-hydroxybutyric acid produced can then be oxidized to acetyl acetate by a dehydrogenase enzyme. Reaction of acetyl acetate with β-ketothiolase generates acetyl coenzyme A which can be used for cell regeneration (Doi and Fukuda 2014; Kobayashi et al. 2005). Several bacteria and fungi, e.g. *Pseudomonas, Actinomadura, Penicillium Aspergillus* spp.*, Microbispora, Saccharomonospora, Streptomyces, Thermoactinomyces* and *Bacillus* spp., all have the ability to degrade PHAs both aerobically and anaerobically. Anaerobic sludge containing several microorganisms can degrade PHAs in different environments such as soil, salt and fresh water. However, soil was found to be the most natural environment for PHAs degradation (Sang et al. 2002; Tokiwa et al. 2009; Boyandin et al. 2012).

In vivo degradation of PHB films inside living organisms resulted in nontoxic metabolites where 3-hydroxybutyrate was produced which naturally exists in blood and thus can be used in implant devices (Lee 1996). It has been reported that the PHB degradation rate could possibly be accelerated by the addition of plasticizers or polymers. On the other hand, hydrophilic additives could also accelerate hydrolysis as a result of high water adsorption (Freier et al. 2000).

PHAs copolymers which could be biodegraded anaerobically have various important agricultural applications such as encapsulation of seeds and fertilizers, biodegradable plastic films for crop protection and biodegradable containers for hothouse facilities. Additionally, such copolymers may be used in the coating of herbicides and insecticides (Verlinden et al. 2007; Yogesh et al. 2012). In this study, we report the biodegradation process for various formulations of polyhydroxybutyrate films by soil as part of our ongoing interest in the field of polymeric chemistry (Smith et al. 2011, 2012, 2015; Balakit et al. 2015; Yousif et al. 2015a, b).

## Methods

### Production and recovery of PHB

The biodegradable PHB polymeric material was obtained as previously reported (Altaee et al. 2015) using *Rhodococcus equi* in the presence of crude palm kernel oil (CPKO) as a carbon source based on the one stage cultivation method.

### Preparation of PHB films

Conventional solvent-cast technique (Sridewi et al. 2006) was used for PHB and PHB–TiO_2_ composite films preparation. The PHB films were prepared by dissolving the extracted polymer (0.3 g) in chloroform (30 ml) in a Schott bottle with magnetic stirring for 30 min. The mixture was poured into glass petri dishes (9 cm in diameter) as the casting surface. The petri dishes were covered with puncture aluminium sheets and left in the dark at 30 °C for 24 h to allow complete evaporation of chloroform. The commercial TiO_2_ powder (P25 Degussa GmbH, Marl, Germany) was used for the composite preparation in a similar manner to the conventional solvent-casting method where a mixture of PHB (0.3 g) and TiO_2_ powder (0.18 g) was suspended in chloroform (30 ml).

PHB nanofiber films were prepared by an electrospinning technique using Esprayer ES-2000 (Fuence, Co. Ltd., Japan). PHB (0.2 g; 4 % by w/v) was dissolved in a mixture of chloroform and dimethylformamide (5 ml in the ratio of 4:1 by volume). The electrospinning for the solution was carried out at an extrusion rate of 40 µl/min and a voltage of 15 kV. The mixture was stirred for 2 days at room temperature, to ensure complete homogeneity, followed by stirring at 55 °C for 2 h. The solution was loaded into a glass syringe (1 ml maximum loading) equipped with a stainless steel needle (0.5 mm in diameter). The distance from the needle tip to the collector was fixated at 20 cm. The copper collecting plate was covered with insulating material, leaving a circular hole (5 cm diameter) for deposition of the resultant fibre. PHB–TiO_2_ composite nanofiber films were prepared in a similar manner of electrospinning by the addition of TiO_2_ powder (0.12 g) to PHB in mixed solvent (Sudesh 2013).

Other PHB films were prepared by the conventional solvent-cast technique (Sridewi et al. 2006) and treated for 24 h under a UV light (30 W) source with a 5 cm distance. After treatment, such films were used as a source for PHB in the preparation of PHB and PHB–TiO_2_ composite films using the method used for the preparation of PHB and its composite. Since TiO_2_ is photosensitive, all the Schott bottles used for the preparation of nanocomposite films using the conventional solvent-cast and the electrospinning techniques were wrapped with aluminium foil and kept in a dark place before use. Also, all cast films were aged for one week to reach equilibrium crystallinity, before subjecting them for degradation.

### Biodegradation of films in soil

The site chosen to carry out the degradation study was a fertile garden with pH 7.30 and humidity of 80 % at 30°C (University Science of Malaysia, USM). The films were cut into pieces (1 cm × 1 cm) and placed inside none degradable mesh bags (8 cm × 4 cm) where each bag was divided into smaller pouches (1.5 cm × 1.5 cm). All film samples were prepared in triplicate with weighting and sealed by non-degradable thread. The mesh bags were fixed on a metal mesh and buried in soil 10 cm from the surface for 6 weeks.

### Determination of molecular weight of film samples

The molecular weights of film samples were determined before and after the degradation. The molecular weights of the extracted and purified polymers were determined by an Agilent 147 1200 gel permeation chromatography (GPC; Agilent, CA, USA) connected to a refractive index detector with 148 Shodex K-806 (Agilent, CA, USA) columns. Polymers (1 mg/ml) were dissolved in chloroform (HPLC grade 149) and filtered through 0.45 µm PTFE membrane. Chloroform was also used as the eluent with a flow rate of 0.8 ml/min at 40 °C. Universal calibration was generated using a narrow dispersity polystyrene standard (Agilent, CA, USA).

### Measurement of biodegradation percentage by weight loss

Each week, one mesh bag which contained all of the film samples was taken out and washed with sterile distilled water to remove any soil residual particles and was left to dry at room temperature for 24 h. The dried samples were placed in a desiccator for an hour to allow it to reach the constant weight. The weights of samples were then recorded and the degradation percentage was calculated as a function of weight loss using Eq. 1 (Yew et al. 2006).

1 $${\text{Degradation}}\,\% = \left[ {\left( {W_{1} - W_{2} } \right)/W_{1} } \right] 100$$

where *W*_1_ is the initial weight of the film and *W*_2_ is the weight of the film after degradation.

### Quantitative microbial counting

The standard spread plate technique was used to measure the microbial growth in the soil of the buried and near to the samples every week. The medium used was tryptic soy agar (TSA). The soil obtained (1 g) was washed using sterilized normal saline solution (99 ml, 0.95 %) with gentle mixing by a vortex followed by serial dilution and decantation. Each dilution (1 ml) was spread onto TSA plates in triplicate for each dilution. The plates were incubated at 30 °C for 48 h and the colonies (30–300) were examined and counted to measure colony-forming unit (CFU) for each sample (log_10_ CFU/ml).

### Microscopic observation of surface changes after degradation

The changes in surface morphology of the films were checked every week by Olympus S240 Stereo Microscope (Olympus, Tokyo, Japan) fitted with a JVC K-F55B colour video camera. Each sample (5 mm × 5 mm), fixed on aluminium stumps, coated with gold for 15 s and viewed under scan electron microscope (SEM; Carl-Ziess SMT, Oberkochen, Germany) at an acceleration voltage of 5 kV.

### Statistical and data analysis

The degradation data was analyzed with a completely randomized design using the least significant difference (LSD) at a significant level of 0.05 (Al-Rawi and Khalaf Allah 2000).

## Results and discussion

All PHB films were prepared by the casting method, but the nanofiber films were prepared by electrospinning. After incubation time of degradation, the changes in molecular weight (Mw), the number-average molecular weight (Mn) and the dispersity (Mw/Mn) for all polymeric films were measured by GPC. The degradation of all films was associated with a significant decrease in MW, Mn and Mw/Mn ratio. PHB films showed significant decreases in Mw and Mn, while, PHB nanofiber and PHB–TiO_2_ composite nanofiber films showed significant decreases in Mw/Mn ratio. The decreases in Mw, Mn and MW/Mw ratio can be attributed to biodegradation of polymer samples due to enzymatic activity of living organisms in which CO_2_ and H_2_O was produced under aerobic conditions and CO_2_, H_2_O and methane under anaerobic conditions (Avella et al. 2005). The results obtained are recorded in Table 1.

**Table 1:** The effect of soil degradation on the different type of PHB films after 6 weeks

Treatment/films	After treatment^a,b^			Before treatment^a,b^		
	Mw/Mn	Mn	Mw	Mw/Mn	Mn	MW
PHB	1.54 ± 0.11	275 ± 0.06	396 ± 0.19	1.72 ± 0.13	373 ± 0.02	642 ± 0.45
PHB–TiO_2_	1.73 ± 0.10	352 ± 0.12	609 ± 0.56	1.82 ± 0.07	370 ± 0.07	674 ± 0.33
PHB nanofiber	1.32 ± 0.03	301 ± 0.07	538 ± 0.81	1.79 ± 0.15	421 ± 0.16	554 ± 0.02
PHB–TiO_2_ nanofiber	1.41 ± 0.15	350 ± 0.01	495 ± 0.71	1.78 ± 0.46	389 ± 0.09	624 ± 0.14
PHB (UV)	1.56 ± 0.53	306 ± 0.02	476 ± 0.12	1.79 ± 0.01	339 ± 0.02	606 ± 0.02
PHB–TiO_2_ (UV)	1.39 ± 0.04	314 ± 0.02	435 ± 0.15	1.78 ± 0.03	335 ± 0.02	597 ± 0.06

^a^The average and the standard deviation were measured from three parallel studies

^b^LSD is at 0.5 level for interaction before and after degradation time for all films types (Mw = 12.301, Mn = 10.312, Mw/Mn = 0.042)

**Fig. 1:**
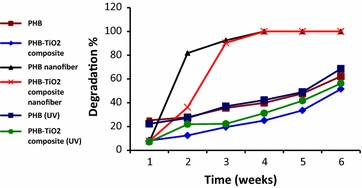
The degradation percentage of PHB films up to 6 weeks

On the other hand, the degradation percentage for all of the film types were increased as the incubation time of degradation increases. The PHB nanofiber and PHB–TiO_2_ composite nanofiber films showed the highest degradation percentage compared to the other films. Complete degradation of nanofiber films was achieved after 3 weeks (Fig. 1). This remarkable degradation of nanofibers could be due to the large surface area, high porosity level and its three dimensional structure which allowed a greater mass of microorganisms to attach to the polymeric films (Gupta et al. 2005). The PHB film prepared from PHB films that have been treated by ultraviolet (UV) light and untreated PHB sample showed a weight loss of ~68 and 62 %, respectively. While, PHB–TiO_2_ composite films and the one prepared from PHB films treated by ultraviolet (UV) light showed low weight loss (~51 and 56 %, respectively). The low degradation percentage could be due to the reduction in antibacterial growth by the presence of TiO_2_ (Ahmad and Sardar 2013; Verdier et al. 2014).

**Fig. 2:**
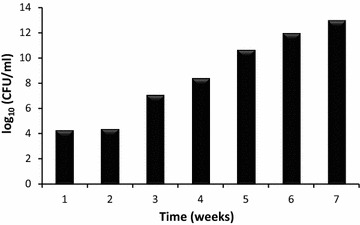
The microbial number in soil at the buried site for different PHB films

The number of microorganisms in soil at the buried site was expressed as log_10_ CFU/ml (Fig. 2). The microbial population increased with incubation time which led to an increase in polymer degradation. The soil microbes can exert depolymerase enzymes that can hydrolyse PHB polymers and utilities the metabolic degradation products as a source of energy and nutrients (Doi 1990; Kumaravel et al. 2010).

**Fig. 3:**
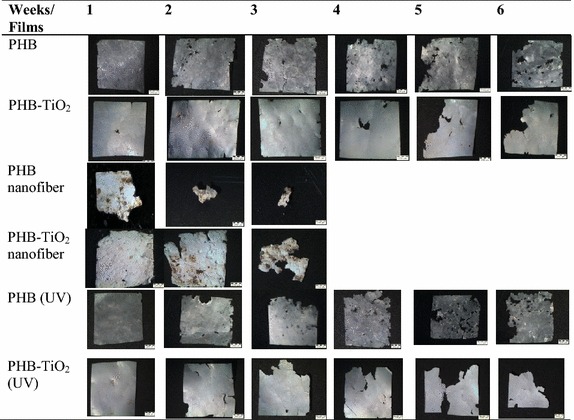
The physical changes in polymeric films due to soil degradation

The physical changes in surface morphology of polymeric sheets were detected by the use of a light microscope (Fig. 3). Such changes include cracks, holes, gradual loss of parts and color changes. PHB nanofiber and PHB–TiO_2_ composite nanofiber films were found to be completely degraded after 3 weeks. It was noted that there was a correlation between the weight loss percentage and the physical changes in surface morphology (Figs. 1, 3). This correlation was shown to increase week after week. The degradation of PHB nanofiber and PHB–TiO_2_ composite nanofiber was complete after 3 weeks where the weight loss was 100 %. However, the incorporation of TiO_2_ in the films led to a reduction in biodegradation when compared to the PHB films, this could be possibly due to TiO_2_ inhibiting microbial growth (Haghi et al. 2012; Verdier et al. 2014). The PHB and its composite films prepared from PHB films treated by ultraviolet (UV) light were found to have more cracks when compared to those where prepared from the PHB films without UV treatment. It therefore clear that UV treatment played a role at accelerating the degradation process which is consistent with the results reported by Shangguan for the biodegradation of poly(3-hydroxybutyrate-co-3-hydroxyhexanoate) (Shangguan et al. 2006).

**Fig. 4:**
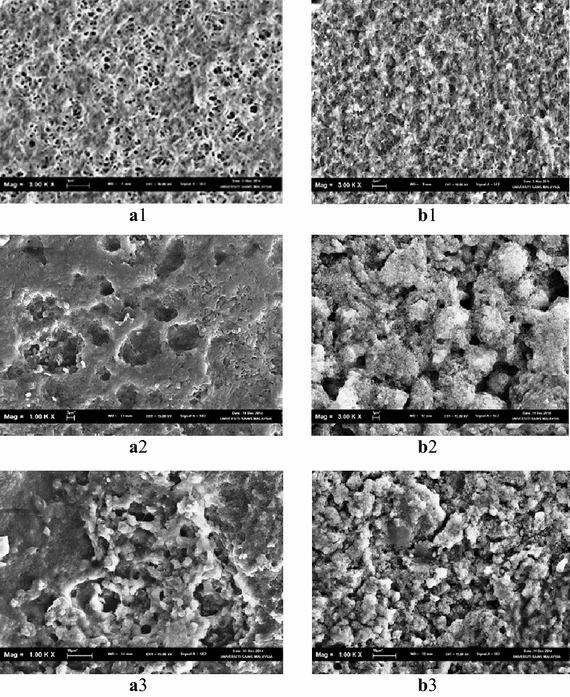
SEM micrographs for PHB and PHB–TiO_2_ composite films. **a1** PHB before degradation, **b1** PHB–TiO_2_ before degradation, **a2** PHB after degradation, **b2** PHB–TiO_2_ after degradation, **a3** PHB after degradation, **b3** PHB–TiO_2_ after degradation

The SEM micrographs for the PHB and PHB–TiO_2_ composite films are shown in Fig. 4 and confirmed various changes that had taken place within the surface of polymer sheets. After the degradation inanimate spherical objects were noted.

**Fig. 5:**
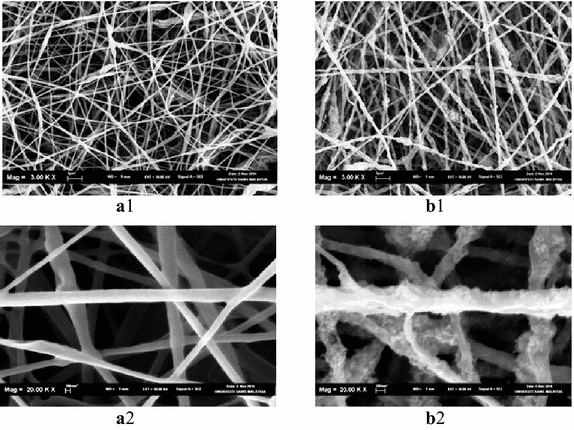
SEM micrographs for PHB and PHB–TiO_2_ nanofiber films before degradation. **a1** PHB before degradation, **b1** PHB–TiO_2_ nanofiber before degradation, **a2** PHB before degradation, **b2** PHB–TiO_2_ nanofiber before degradation

Figure 5 showed the surface morphology for PHB nanofiber (~500 nm diameter) and PHB–TiO_2_ composite nanofiber films (~550 nm diameter) before incubation.

**Fig. 6:**
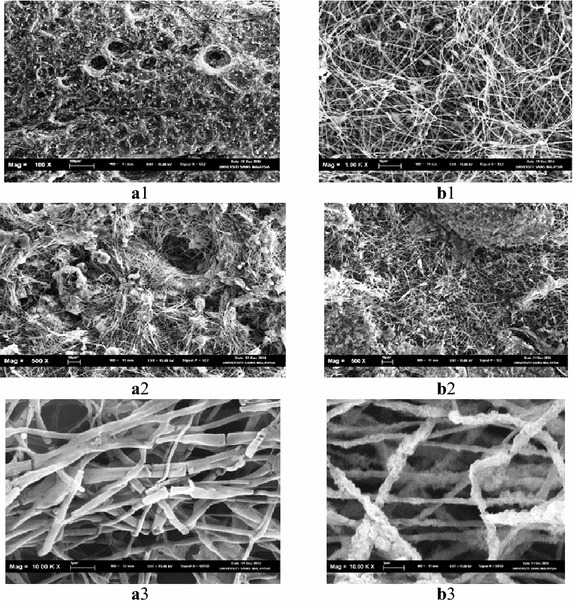
SEM micrographs for PHB and PHB–TiO_2_ nanofibers after degradation. **a1** PHB after degradation, **b1** PHB–TiO_2_ nanofibers after degradation, **a2** PHB after degradation, **b2** PHB–TiO_2_ nanofibers after degradation, **a3** PHB after degradation, **b3** PHB–TiO_2_ nanofibers after degradation

Figure 6 showed the nanofibers films surface after degradation. Clearly, it indicates a non-rank surface with pores, cavities, grooves, extended objects like hyphae of fungi, spherical objects like bacteria, visible ruptures of most nanofibers. Also, it was evident that the nanofibers diameters decreased after degradation to be about 400 nm for PHB nanofiber films and around 480 nm for PHB–TiO_2_ composite nanofiber films. This means that the nanofibers became thinner after degradation compared to the corresponding ones before degradation.

The three dimensional structures and large surface area of nanofibers accelerated the degradation of PHB nanofibers. Even though PHB–TiO_2_ composite nanofibers were faster at degradation compared to the other samples, it was however, less efficient than PHB nanofibers. Clearly, the addition of TiO_2_ nanoparticles leads to the reduction in the antibacterial activity. Similar results for the surface morphology changes were also reported for poly(hydroxybutyrate-co-hydroxyvalerate), PHBV and their composite (Buzarovska et al. 2009).

**Fig. 7:**
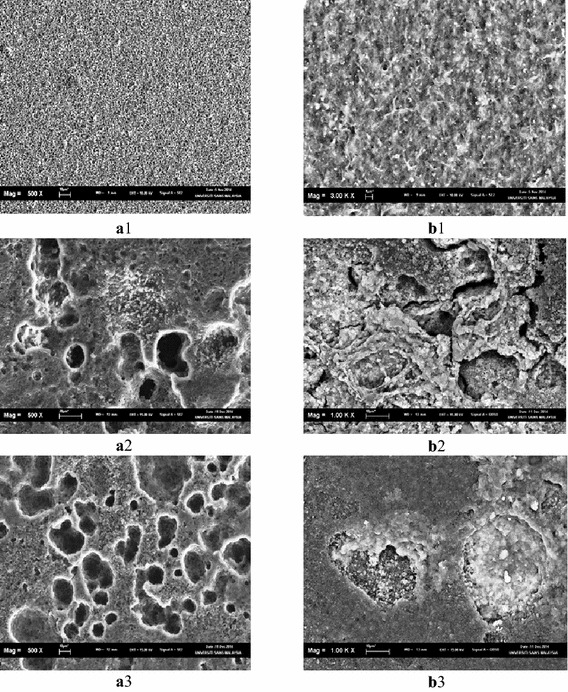
SEM micrographs for PHB and PHB–TiO_2_ films that prepared from UV treated PHB films. **a1** PHB (UV) before degradation, **b1** PHB–TiO_2_ (UV) before degradation, **a2** PHB (UV) after degradation, **b2** PHB–TiO_2_ (UV) after degradation, **a3** PHB (UV) after degradation, **b3** PHB–TiO_2_ (UV) after degradation

Figure 7 showed the SEM micrographs for the PHB film and its composite that have been prepared from UV treated PHB films. Clearly, more apparent physical changes have taken place within the polymeric surface compared to the ones which involved no UV treatment. Such changes are clear signs of degradation of polymer film samples.

## Conclusions

Biodegradation of PHB is of great interest due to the increased usage of PHB polymers in agriculture to overcome pollution problems associated with the petroleum polymers handling. The degradation of different PHB formulations and their composite films have been studied in fertile soil. The degradation was evaluated by measuring microbial growth, polymeric material weight loss and physical changes within the surface of polymeric films through the use of SEM micrograph. All types of polymeric films were degraded to monomers and oligomers of *R*-3-hydroxybutyrate which are subsequently assimilated by microorganisms and their enzymatic activities. It has been found that PHB nanofiber and their TiO_2_ composite were degraded faster compared to other PHB film types as a result of their three dimensional structures and large surface area. The presence of TiO_2_ nanoparticles in the composite films slowed down the degradation process when compared to PHB films. Finally, the PHB and its composite films which were treated with UV led to faster degradation.
